# The substitute ENSO 16 has low impact on glucose metabolism in healthy humans: a randomized, double-blind, active-controlled, cross-over trial

**DOI:** 10.1038/s41598-024-65560-w

**Published:** 2024-06-24

**Authors:** Martin Lutnik, Stefan Weisshaar, Lena M. Mussbacher, Daniel Steiner, Michael Wolzt

**Affiliations:** 1 Department of Clinical Pharmacology, Medical University of Vienna, Vienna, Austria; 2 NEOH Alpha Republic GmbH, Rooseveltplatz 11/1, 1090 Vienna, Austria; 3 Department of Hematology and Hemastaseology, Medical University of Vienna, Vienna, Austria

**Keywords:** Sugar substitute, Glucose metabolism, Dietary products, Natural sweeteners, Gastric symptoms, Plant fibers, Dietary carbohydrates, Metabolism

## Abstract

High sugar consumption is associated with cardiovascular diseases and diabetes. Current sugar substitutes may cause taste sensations and gastrointestinal symptoms. ENSO 16 is a combination of 16 different sugar substitutes and plant fibers and has been designed as a sugar alternative. The impact on plasma glucose metabolism as well as on gastrointestinal tolerance has not been investigated yet. 17 healthy participants were enrolled in this randomized, double-blind trial. Participants received a single oral dose of 30 g glucose or 30 g ENSO 16 and crossed over to the alternate treatment after a 7 day wash out period. The study endpoint was the effect on plasma glucose, insulin, C-peptide concentrations and gastrointestinal disorders. A questionnaire regarding gastrointestinal symptoms was used for individual subjective scoring. The mean baseline adjusted plasma glucose AUC_0–180 min_ was significantly greater after glucose administration compared to ENSO 16 (n = 15, *p* = 0.0128, paired t-test). Maximum plasma glucose elevation over baseline was 117 mg*dl^−1^ and 20 mg*dl^−1^ after oral glucose or ENSO 16, respectively. Insulin and C-peptide AUC_0−180 min_ were significantly greater after glucose compared to ENSO 16 intake (*p* < 0.01, Wilcoxon rank sum test). The mean maximal concentrations of plasma glucose, insulin and C-peptide after glucose intake were 1.5, 4.6 and 2.7-fold greater after glucose intake compared to ENSO 16 intake, respectively. Adverse reactions were mostly mild and not different between treatments. Conclusion. ENSO 16 has only a small impact on plasma glucose metabolism. This may be of interest in a dietary context and may help to reduce calory intake.

*Trail registration* NCT05457400. First registration: 14/07/2022. https://clinicaltrials.gov/study/NCT05457400.

## Introduction

Elevated intake of glucose and/or fructose may confer an increased risk of diseases such as diabetes mellitus, obesity, dental caries, and cardiovascular diseases^1–5^. Frequent consumption of sugary products or high carbohydrate food is also associated with insulin resistance and the development of non-alcoholic fatty liver disease (NAFLD)^6^.

The World Health Organization advocates that added sugars should constitute < 10% of daily caloric intake, and there is a discussion to reduce this recommendation further^2^. Consequently, there has been increased research for alternative sugar substitutes. Sweeteners are broadly categorized into two main groups: non-caloric high-intensity sweeteners and nutritive sweeteners. The first group includes a variety of synthetic sweeteners, such as advantame, acesulfame-K, aspartame, neotame, saccharin, and sucralose. The group of nutritive sweeteners includes sugar alcohols such as erythritol, lactitol, mannitol, maltitol, sorbitol, trehalose, and xylitol. Sugar alcohols, overall, possess a slightly lower caloric content than table sugar and do not contribute to tooth decay, as they are not fermentable by oral bacteria. The sweetness of sugar alcohols ranges from 25 to 100% of the sweetness level of sucrose. These sugars serve primarily as bulking agents in artificial sweeteners and are commonly found in "sugar-free" products^4,7–9^. Aspartame tastes around 200 times sweeter than glucose and frequently used as sweetener in dietary products^10^. However, its safety is under debate and the substance has been labeled as "possibly carcinogenic to humans" recently^11^.

In general, sugar substitutes are associated with issues of gastrointestinal tolerability, taste sensations, including bitterness, and a cool aftertaste^9^. While inulin and oligofructose may exert a prebiotic effect, excessive consumption of these substances is laxative and may cause severe flatulence. Erythritol has attracted interest because it does not impact glucose and insulin concentration^10,12^. Plant fibers as substitutes may provide protection against cardiovascular diseases, type 2 diabetes, and colorectal and breast cancer^13^.

There is a lack of experience how a combination of sweeteners interacts with glucose metabolism. ENSO 16 is a mixture of 16 different sugar substitutes and plant fibers. The formulation was developed to produce a sugar substitute that, in contrast to other sweeteners and sugar substitutes, does not have an unpleasant taste. In addition, due to the composition of substrates at low doses and the low energy density compared to glucose, ENSO 16 should have only minor if any effects on plasma glucose concentration. However, this has not been demonstrated yet under appropriately controlled conditions.

The aim of the study was to evaluate the metabolic impact of ENSO 16 on plasma glucose, insulin and C-peptide concentrations and to evaluate its tolerability in healthy humans in comparison to a standard dose of oral glucose.

## Methods

This trial was designed as a randomized, double-blind cross-over study. The effect of the sugar substitute ENSO 16 was compared with a 30 g glucose in water solution on glucose metabolism parameters in healthy male or female humans. The trial was approved by the Ethics Committee of the Medical University of Vienna (EK 1126/2022). The study was conducted in accordance with the protocol, the principles of the "Declaration of Helsinki" in its current version and the requirements of the Austrian law (AMG). According to the stipulations of the Austrian Data Protection Law, confidentiality and pseudonymity of the volunteers were assured. The informed consent was obtained from all the participants for study participation. The recruiting and data allocation took place between 11. July 2022 and 29. July 2022.

### Population

18 healthy participants (7 males, 8 females) were included in the study. Participants that dropped out were replaced according to protocol to generate data of 15 different individuals. Inclusion criteria were signed informed consent, age > 18 years, body mass index 18–25 kg*m^−2^ and a fasting capillary plasma glucose concentration ≤ 100 mg*dL^−1^. Exclusion criteria were presence of any chronic illness, smoking or drug abuse, food allergies, pregnancy or lactations or participation in another trial 3 weeks before the first study day.

### Study product

The investigational product ENSO 16 (NEOH by Alpha Republic GmbH, Rooseveltplatz 11/1, 1090 Vienna, Austria) is a sugar substitute mixture containing 62% organic fibers like inulin and oligofructose and 38% of other sugar alcohols and sweetener substances. The composition is confidential and not fully disclosed. ENSO 16 was produced, packaged and labeled by STAMAG Stadlauer Malzfabrik GesmbH, Smolagasse 1, 1220 Vienna, Austria according to applicable food law provision and guidelines. The active comparator was glucose powder provided by Special Ingredients Ltd, 4, Foxwood Industrial Park, Chesterfield, S41 9RN, UK. 30 g of each substance were dissolved in 200 mL water for oral administration. A similar amount of sweetener was used in other investigations of sugar substitutes to describe endocrine responses^14^. 30 g of ENSO 16 contain 198 kJ (49.5 kcal) with an amount of 9.3 g carbohydrates of which 0.72 g is glucose. 30 g of glucose have an equivalent of 486 kJ (116 kcal). The FDA recommends a maximum daily intake of added sugar of 200 kcal (48 g).

### Study procedure

Two study days with a washout period of at least 7 days were planned. Participants were randomized to receive ENSO 16 or glucose on the first study day. The sponsor conducted randomization using a predefined randomization list from the web application at www.randomizer.org. The study medication was provided by the sponsor in identical-looking bottles. Due to their completely identical appearance and smell (white, powdery substance, sweet smell), no difference could be perceived by the investigators or subjects. Preparation of the study product on the study days was performed by blinded investigators. Unblinding was performed after the database lock. Following the wash out period, subjects crossed over to the alternate treatment on the second study day. (Supplement Table 1) Plasma samples for analysis of glucose, C-peptide and insulin were collected under fasted conditions and at pre-defined timepoints (15, 30, 45, 60, 90, 120, 180 min) after single administration of ENSO 16 or glucose. (Supplement Fig. 1) Gastrointestinal symptoms (abdominal pain, nausea, vomiting, diarrhea, abdominal rumbling, bloating, belching and flatulence) were assessed using a standardized questionnaire. Participants were asked to choose between “no symptom” (0 points), “mild symptoms” (1 point) and “severe symptoms” (2 points) for each question at each time point. (Supplement Table 2).

**Table 1:** Baseline characteristics of the study population, n = 15.

Baseline characteristics						*p* values
Female		n, %	8		53.3	
Male		n, %	7		46.7	
BMI	[kg*m^−2^]	Mean ± SD	22	±	2	
Age	[yrs]	Mean ± SD	28.4	±	6.1	
Fasting glucose D1	[mg*dL^−1^]	Mean ± SD	87	±	8	
Fasting glucose D2	[mg*dL^−1^]	Mean ± SD	89	±	7	0.3746 vs. Day 1

**Figure 1:**
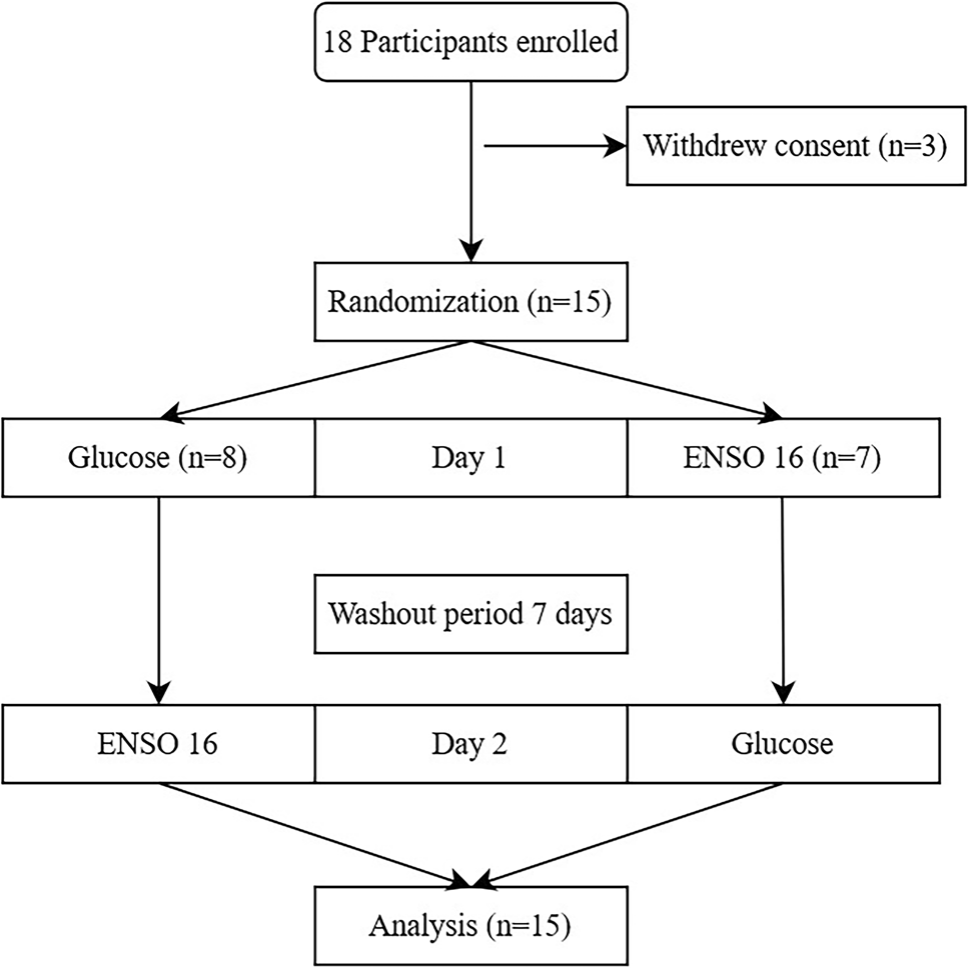
Study flow chart.

**Table 2:** Fasting plasma concentrations of glucose, insulin and C-peptide at baseline in participants receiving a single dose of glucose or ENSO 16.

	Glucose	ENSO 16	*p* values
Glucose [mg*dL^−1^]	79 ± 9	80 ± 6	0.5069
Insulin [µIU*mL^−1^]	6.36 ± 2.09	6.59 ± 2.08	0.676
C-peptide [ng*dL^−1^]	1.78 ± 0.69	1.67 ± 0.35	0.4593

Results are mean ± SD, n = 15 per treatment. Differences in laboratory values were not significant between treatments at baseline (Student’s paired t-test).

### Methodology

Plasma glucose, insulin and C-peptide concentrations were analyzed according to laboratory standards at the Department of Laboratory Medicine, Medical University of Vienna.

Venous plasma sampling was performed from an indwelling cannula in an antecubital vein. To assess plasma glucose concentrations, blood was drawn into tubes containing sodium fluoride K3EDTA (Vacuette, Greiner Bio-One, Austria). Insulin and C-peptide concentrations were quantified from blood collected into serum tubes with clot activator (Vacuette, Greiner Bio-One, Austria).

### Study endpoints

Changes in plasma glucose were compared from baseline-adjusted area under the plasma glucose versus time curve until 180 min (AUC_0–180 min_). Secondary endpoints included plasma glucose, plasma insulin and C-peptide, respectively. Further, the impact of ENSO 16 and glucose on gastrointestinal symptoms were assessed.

### Statistics

Due to a lack of data for the impact of ENSO 16 compared to glucose on plasma, a difference of 10% between groups is assumed to be clinically relevant. With a sample size of 15 subjects, based on other trials and recommendations^13^ (cross over) a difference of at least 10% between AUCs (with a standard deviation of 10%) can be detected with 80% power using a paired t test at a significance level of 5% (two-sided). All statistical analyses were conducted using the commercially available GraphPad Prism 9 (GraphPad, 225 Franklin Street. Fl. 26 Boston, MA 02110, USA). Continuous variables were summarized as mean ± standard deviation (SD) if their distributions were consistent with normality. If the distributions were not consistent with normality, the variables were summarized by their median and the 25th to 75th percentile range. Between-group comparisons for primary and secondary outcome parameters were assessed using paired t-tests or Wilcoxon signed rank tests as appropriate. Differences were considered significant if the p-value was less than 0.05. For the evaluation of insulin, C-peptide, and glucose profiles, baseline-corrected values were used to calculate the area under the curve (AUC_0–180 min_) using the trapezoidal rule. Maximum plasma concentrations were used to calculate individual ratios of effects between treatments.

### Ethical approval

The trial was approved by the Ethics Committee of the Medical University of Vienna (EK 1126/2022).

## Results

18 participants were screened to include 15 subjects (7 male, 8 female). Two female and one male study participants withdrew consent before treatment. The study flow chart is shown in Fig. 1. Baseline characteristics of study population are shown in Table 1. Baseline concentrations of glucose metabolism parameters of both treatment groups are presented in Table 2. There were no differences in fasting glucose concentration between the two study days or between treatment groups at baseline, respectively. There were no study discontinuations due to poor tolerability.

### AUC of plasma glucose, insulin and C-peptide (Table 3)

**Table 3:** Baseline-adjusted area under the concentrations versus time curve [AUC_0–180 min_] of plasma glucose, insulin and C-peptide after single oral intake of glucose or ENSO 16.

	Glucose	ENSO 16	*p* value
Glucose AUC_0–180 min_ [mg*dL^−1^*min]	1258 ± 2002	− 85 ± 593	0.0128
Insulin AUC_0–180 min_ [µIU*mL^−1^*min]	1509.70 ± 833.30	− 44.06 ± 325.60	< 0.01
C-peptide AUC_0–180 min_ [ng*dL^−1^*min]	252.30 ± 142.50	9.50 ± 27.70	< 0.01

Results are mean ± SD, n = 15 per treatment. Differences between treatments are indicated (Student’s paired t-test).

The mean baseline-adjusted AUC_0–180 min_ after glucose was significantly greater compared to ENSO 16 (*p* = 0.0128, paired t-test). Insulin and C-peptide baseline adjusted AUC_0–180 min_ were significantly greater after glucose compared to ENSO 16 intake (*p* < 0.01, Wilcoxon rank sum test) (Supplement Fig. 2).

**Figure 2:**
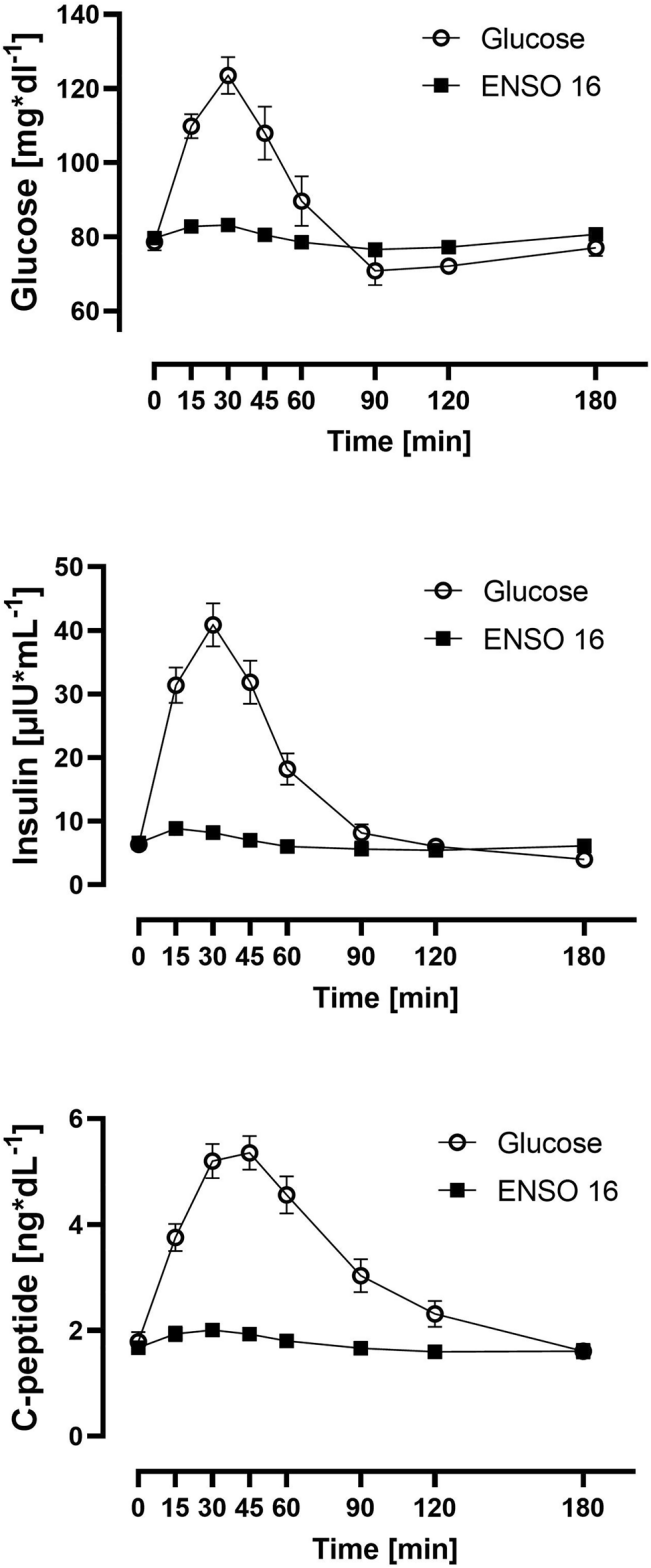
Time course of plasma glucose, insulin and C-peptide concentration after single oral intake of glucose (open circles) or ENSO 16 (solid squares). Data are mean ± SEM, n = 15 per treatment.

### Time course of plasma glucose, insulin and C-peptide concentration (Fig. 2)

#### Plasma glucose concentration

Oral administration of glucose or ENSO 16 increased plasma glucose concentrations, which was different between treatments.

The mean maximal plasma glucose concentration was 124 ± 18 mg*dl^1^, with a maximum individual concentration of 187 mg*dl^−1^ at 45 min after glucose intake. The mean relative increase of plasma glucose was 45 ± 20 mg*dl^1^, with maximal relative increase of 117 mg*dl^1^ over baseline.

In contrast, the mean maximal plasma glucose concentration after ENSO 16 reached a maximum of 83 ± 5 mg*dl^1^ after 15 min, with a maximum individual concentration of 92 mg*dl^−1^. The mean relative increase of plasma glucose was 4 ± 4 mg*dl^1^, with a maximum relative increase of 20 mg*dl^−1^ over baseline.

The mean ratio of the individual maximum plasma glucose concentration after glucose intake was 1.5-fold greater than after ENSO 16 intake.

#### Insulin concentration

After glucose intake, insulin increased about sixfold from baseline to a mean maximum of 40.9 ± 12.7 µIU*mL^−1^ after 30 min, with a maximal relative increase of 69.5 µIU*mL^−1^. After ENSO 16 intake, insulin increased 1.3-fold from baseline to a mean maximum of 8.83 ± 2.42 µIU*mL^−1^ after 15 min, with a maximal relative increase of 4.42 µIU*mL^−1^.

The mean ratio of the maximum plasma insulin concentration after glucose intake was 4.6-fold greater than after ENSO 16 intake.

#### C-peptide concentration

After glucose intake, C-peptide increased about 3-fold from baseline to a mean maximum of 5.35 ± 1.19 ng*dL^−1^ after 45 min, with a maximal relative increase of 3.57 ng*dL^−1^. After ENSO 16 intake, C-peptide increased 1.2-fold from baseline to a mean maximum of 2.01 ± 0.42 ng*dL^−1^ after 15 min, with a maximal relative increase of 0.8 ng*dL^−1^.

The mean ratio of the maximum plasma C-peptide concentration after glucose intake was 2.7-fold greater than after ENSO 16 intake.

### Tolerability

Detailed symptoms are summarized in Table 4. The most frequently reported gastrointestinal symptom was belly growling, with 9 occurrences in the ENSO 16 group and 7 in the glucose group. Diarrhea was reported once in each group, and it pertained to the same participant. Bloating was reported twice in both groups. Other symptoms (abdominal pain, nausea or flatulence) were single occurrences in the ENSO 16 group. One symptom, belly growling, was reported as severe in the ENSO 16 group, and all other symptoms were mild.

**Table 4:** Gastrointestinal symptoms after single oral intake of glucose or ENSO 16.

Symptoms	Glucose	ENSO 16
Abdominal Pain	0	1
Nausea	0	1
Vomiting	0	0
Diarrhea	1	1
Belly growl	7	9
Bloating	2	2
Burping	0	0
Flatulence	0	1

Absolute occurrences of symptoms in study participants are presented, n = 15.

## Discussion

In this randomized controlled trial, a single oral intake of the sugar substitute ENSO 16 resulted in a substantially lower plasma glucose, insulin and C-peptide response compared to glucose intake. Gastrointestinal tolerability of ENSO 16 was at the level of glucose as active comparator.

In our study plasma glucose concentration reached their maximum values 30 min after glucose and 15 min after ENSO 16. The time course and magnitude of changes in glucose metabolism to 30 g oral glucose solution was as expected in the healthy humans under study. In healthy patients, C-peptide and insulin concentrations typically peak at 30–60 min after intake of glucose, which agrees with our study. In contrast, in patients with glucose intolerance or type 2 diabetes peak plasma glucose concentrations are delayed up to 120 min. Furthermore, plasma glucose and insulin concentrations are higher than in a healthy population^15,16^. Insulin enters the bioactive state through the decoupling of C-peptide from proinsulin. C-peptide has a longer half-life than insulin^15^. This is consistent with the observed peak of C-peptide after 45 min, which occurred after the insulin peak after glucose intake. Following ENSO 16, the changes in C-peptide and insulin over time were only small. Oral intake of food may provoke a cephalic-phase insulin release induced by sensory signals before elevation of blood glucose levels to prepare for glucose uptake^17^. We did not test for such an effect in our investigation as this phenomenon would be expected to be equally in both groups due to the blinded setting.

It is generally known that high and rapid glucose intake can cause rebound hypoglycemia due to the insulin response after administration. In our study, there were no occurrences of hypoglycemia. However, plasma glucose concentrations were lower after 180 min compared to baseline in 10 participants after glucose and 3 participants after ENSO 16, which indicates that the selected dose was a physiologically relevant challenge in healthy humans. The pronounced insulin response might also be responsible for the occurrence of stomach growling, following the rapid decline in plasma glucose concentration after the early peak as a typical may trigger of an appetite signal^18^.

A potential advantage of ENSO 16 over other sugar substitutes could be its composition from vegetable dietary fibers and other alternative sweeteners. The inclusion of dietary fibers not only contributes to the substance's texture and mouthfeel but may imply potential health benefits. A meta-analysis from 4653 patients has demonstrated a relative risk reduction of 15–30% for clinical outcomes such as type 2 diabetes mellitus or coronary heart for a consummation of high amounts of dietary fiber (15–29 g per day)^13^. Dietary fibers interact with the gut microbiome and its metabolism, which may exert salutary effects on the gastrointestinal tract^19^. Dietary fibers have been associated with enhanced satiety, improved gastrointestinal health, and mitigation of glucose absorption^20,21^. Plant fibers stimulate the growth of bifidobacterial bacteria in the intestine and are completely fermented by colonic microflora, contributing to energy production with approx. 1–2 g/kcal^22^. This mechanism may contribute to the pattern of plasma glucose observed after ENSO 16, as a substantial proportion of the formulation comprises vegetable dietary fibers.

A relevant subjective issue of many sugar substitutes derive from their taste profile. Individuals accustomed to the taste of natural sugar have described that substitutes lack sweetness or exhibit an undesirable aftertaste^9^. ENSO 16 has shown to mimic natural sugar sweetness in the absence of bitter taste sensations in preceding examinations. This may be the result of the variety of multiple sweeteners combined in ENSO 16, which provides a more nuanced and well-rounded sweetness profile compared to single sugar substitutes at high dose.

Some sugar substitutes, particularly certain sugar alcohols, have been associated with gastrointestinal discomfort such as bloating, gas, and diarrhea when consumed in excess^4^. In this single dose study, ENSO 16 intake was tolerated well and gastrointestinal symptoms occurred only in a few participants. However, the effect of higher doses of ENSO 16 or repeated dosing was not assessed in this trial.

Consumption of sugar substitutes may exert a negative impact on metabolism and cause weight gain and metabolic derailment. Sugar substitutes are often considered unresponsive to glucose homeostasis because they do not consistently elicit post-ingestive responses like caloric sugars. This reduces the release of glucagon like peptide 1 (GLP-1), which leads to a lower feeling of satiety. Furthermore, consumption of sugar alternatives showed a different psychological reaction compared to glucose intake. This may lead to an overconsumption of energy intake and changed behavioral responses^23^. Therefore, a professional dietary consultation is imperative to improve nutritional behavior.

## Strengths and limitations

The major strength of this study is its robust trial design with masked treatment conditions. A limiting aspect is the fact that sweetness experience in combination with food as well as effect of different doses were not tested. Finally, this study only evaluates the effect of ENSO 16 in healthy humans. Further investigation in patients with impaired glucose control and overweight as well as after repeated dosing in combination with other food intake are necessary. Fasting glucose levels were used to investigate a physiological glucose metabolism in volunteers. No carry over effects was detectable between study days. ENSO 16 intake may result in an interaction with concomitant intake of food components. Further investigations are therefore needed to evaluate a potential effect.

## Conclusion

The sugar substitute ENSO 16 showed only a small impact on plasma glucose, insulin, and C-peptide as circulating indicators of glucose metabolism and a good gastrointestinal tolerability. ENSO 16 may have potential beneficial effects in a dietary context.

### Supplementary Information

Supplementary Information.

## Data Availability

The datasets generated and analyzed during the current study are available from the corresponding author on reasonable request.

## Abbreviations

AUC: Area under the concentration versus time curve
SD: Standard deviation
min: Minutes
