# Supplementary material for: The substitute ENSO 16 has low impact on glucose metabolism in healthy humans: a randomized, double-blind, active-controlled, cross-over trial
Source: Sci Rep. 2024 Jun 24;14:14534. doi: 10.1038/s41598-024-65560-w (PMC11196703; doi:10.1038/s41598-024-65560-w)

**Supplementary Material**

**Supplement Table 1**

Visit and Assessment Schedule

|  | Visit 1: | Visit 2:  1-2 weeks after Visit 1 |
| --- | --- | --- |
| Obtain informed consent | X |  |
| Check for inclusion/ exclusion criteria | X |  |
| Assessment of heart rate, blood pressure & fasting blood glucose (point of care device) | X | X |
| Pregnancy test (urine) | X | X |
| Medical history | X |  |
| Collect blood sample at  8 defined timepoints | X | X |
| Questionnaire regarding adverse events of the study medication | X | X |

**Supplement Table 2**

Subjective assessment of gastrointestinal symptoms after oral administration of the investigational products; A questionnaire was used to assess subjective gastrointestinal symptoms of the investigational products after administration of ENSO 16 or glucose. Study participants rated subjectively 8 different symptoms - (a) abdominal pain, (b) nausea, (c) vomiting, (d) diarrhea, (e) abdominal rumbling, (f) bloating, (g) belching and (h) flatulence – with "no symptoms" (0 points), "mild symptoms" (1 point) or "severe symptoms" (2 points) at each time point

|  | 0 min | 15 min | 30 min | 45 min | 60 min | 90 min | 120 min | 180 min |
| --- | --- | --- | --- | --- | --- | --- | --- | --- |
| Abdominal pain |  |  |  |  |  |  |  |  |
| Nausea |  |  |  |  |  |  |  |  |
| Vomiting |  |  |  |  |  |  |  |  |
| Diarrhea |  |  |  |  |  |  |  |  |
| Abdominal Rumbling |  |  |  |  |  |  |  |  |
| Bloating |  |  |  |  |  |  |  |  |
| Belching |  |  |  |  |  |  |  |  |
| Flatulence |  |  |  |  |  |  |  |  |

**Supplement Figure 1**

Study Flowchart


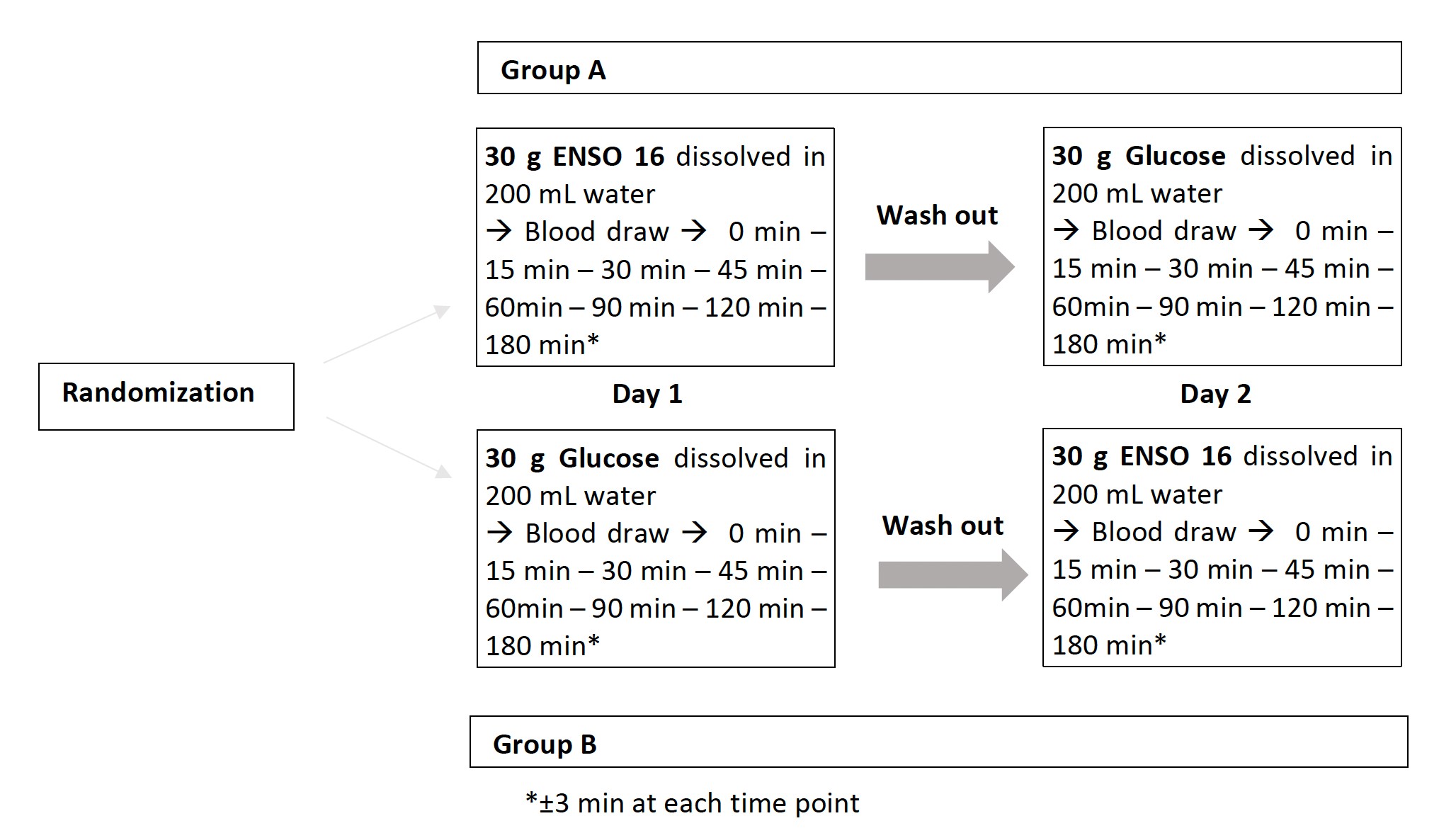


**Supplement Figure 2**

Comparison of baseline adjusted area under the curve 0-180min of glucose, insulin and C-peptide after intake of glucose or ENSO 16; Baseline adjusted AUC(0-180min) of (**A**): plasma glucose levels, (**B**): insulin levels and (**C**): C-peptide levels; n=15 in each cohort; Marked with *: two folded paired T-test, p=0.0128; Marked with **: Wilcoxon rank sum test, p < 0.01;


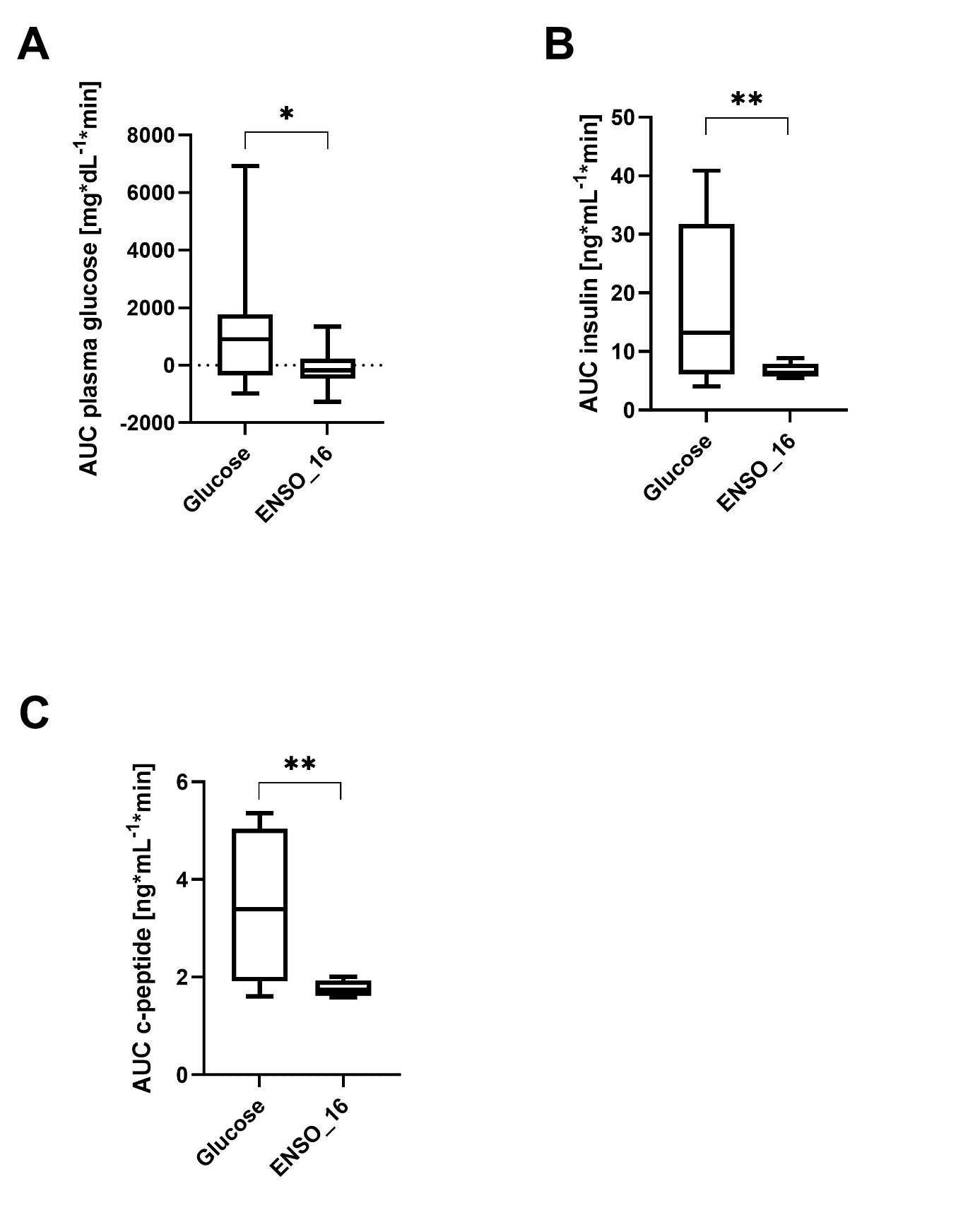

Supplement: Supplementary file 1 — Supplementary Information. [file 41598_2024_65560_MOESM1_ESM.docx]
